# Prevalence of anemia and its associated factors among school-going adolescent girls in schools of Dhankuta municipality, Nepal

**DOI:** 10.1371/journal.pgph.0003684

**Published:** 2024-09-17

**Authors:** Milan Ghimire, Sheetal Bhandari, Manish Rajbanshi

**Affiliations:** 1 B.P. Koirala Institute of Health Sciences, Dharan, Nepal; 2 Central Department of Public Health, Institute of Medicine, Tribhuvan University, Kathmandu, Nepal; Indian Institute of Public Health Hyderabad, INDIA

## Abstract

Adolescent girls are more vulnerable to anemia, especially in low and middle-income countries like Nepal. It is due to early marriage and pregnancy, rapid physical growth, low body weight, economic disparities, heavy menstrual bleeding, and inadequate iron diet. This study aimed to determine the prevalence of anemia and its associated factors among school-going adolescent girls. A cross-sectional study was conducted among 405 adolescent girls using a stratified random sampling technique from both public and private schools. A statistical analysis was performed using IBM Statistical Package for Social Sciences (SPSS) version 25 software. The frequencies, percentages, mean, and standard deviation were used to describe the characteristics of the participants. Binary logistic regression was performed for multivariable analysis. All the tests were performed at a 95% Confidence Interval (CI) and p-value of <0.05. The mean (± SD) age of the participants was 14.2 ± 1.2 years. The majority of the participants (64.7%) were from government schools. Around 18% (95% CI: 13.8, 21.2) of the participants were anemic in the study. Poor knowledge of anemia (AOR = 3.3, CI: 1.0, 11.1), incomplete iron and folic acid intake (AOR = 26.8, CI: 8.3, 86.4), and absence of dietary diversity (AOR = 2.5, CI: 1.1, 9.2) were significantly associated with the higher prevalence of anemia among the adolescent girls. To reduce the risk of anemia among adolescent girls, a comprehensive strategy involving a school health intervention package on anemia, IFA supplements, and deworming program should be promoted. Besides, community-focused awareness programs should be strengthened to increase dietary diversity and improve awareness of anemia in the community.

## Introduction

Anemia is the condition of low hemoglobin (Hb) level in red blood cells and is caused by iron deficiency that results in oxygen-deprived blood, red blood cells, and low oxygen-carrying capacity to the body’s cells [1]. Adolescent girls are more vulnerable to anemia, mainly due to iron deficiency and other contributing factors such as infections, infestations, genetic disorders, early marriage, adolescent pregnancy, rapid growth, low body weight, economic disparities, heavy menstrual bleeding, and inadequate iron intake in the diet [2–4].

Anemia in adolescent girls can detrimentally affect their physical and mental development, affecting education and increasing school drop-out rates. It impairs cognitive development, learning ability, and productivity, increasing infection risks and reducing physical fitness [5–10]. In pregnant adolescents, anemia can result in adverse outcomes such as premature birth, low birth weight, stillbirth, and maternal death.

Globally, the prevalence of anemia is 6% in developed countries and significantly higher in developing countries i.e., 26% [11,12]. Anemia among adolescents is a threat to low-and middle-income countries (LMICs) countries like Nepal and is becoming a significant public health concern [3]. In South Asia, more than half (55%) of adolescent girls suffer from anemia while only 40% of these girls are getting the right amount of nutrients they need through their diet [13].

In Nepal, two of every ten (i.e. 21%) non-pregnant girls aged 10 to 19 years have anemia, Among them, 14% have mild anemia, and 6% have moderate anemia, according to a national survey [8]. The government of Nepal (GoN) has been striving to curve the high burden of anemia through the implementation of anemia control programs such as iron and folic acid supplementation, deworming, and school-based intervention packages including regular screening and treatment of anemic targeting adolescent girls [14–16]. Despite these efforts, the prevalence of anemia among adolescent girls remains high, especially in rural areas of Nepal [9].

It is crucial to address the factors that contributing to anemia among adolescent girls to prevent future maternal and child mortality and morbidity outcomes [17]. Sustainable Development Goal 2.2 (SDG) aims to end all type of malnutrition by 2030, emphasizing nutrition for children, adolescent girls, pregnant/lactating women, and seniors, intersecting with SDG-3 that focus on improved health through adequate and nutritious food [18]. Several studies highlighted that the school-based interventional packages are cost-effective in reducing the prevalence of anemia and increasing the quality of life among adolescents [19–24]. Similarly, these studies revealed that fortifying or supplementing with iron and micronutrients effectively reduces the risk of anemia and enhances hemoglobin levels in children and adolescents. Therefore, this study aimed to determine the prevalence of anemia and its associated factors among school-going adolescent girls in Dhankuta municipality of Nepal. Our study findings can assist decision-makers in choosing the appropriate interventions to tackle anemia in these age groups.

## Materials and methods

### Study design and setting

A cross-sectional study was conducted in Dhankuta municipality from June to October of 2023. Dhankuta municipality is located in the Dhankuta district of the Koshi Province, which is the eastern hilly region of Nepal. There was a total of 19 secondary schools in this municipality, with the number of 1870 adolescent girls (10–19 years) enrolled. Reproductive health indicators in this area were low [19]. Prior to this study, no surveys were conducted related to anemia among adolescent girls in this area.

### Study population

This study included adolescent girls who were between 10–19 years of age and enrolled in selected public and private schools of Dhankuta Municipality. Participants with disabilities related to hearing, mental, speech problems, drop-out students, absentee students, and pregnant adolescents were excluded from the study.

### Sample size and technique

The sample size was calculated using the single population proportion formula n = Z^2^p q/d^2^ [25]_._ The proportion (37.8%) of adolescent girls having anemia was taken from a similar study conducted by Binaya et.al, 2018 [26]. Assuming 95% confidence interval (CI), 5% allowable error, and 10% non-response rate, the final sample size was 405 for this study.

A total of 19 secondary schools fulfilled the study criteria of 10–19 years adolescent girls in Dhankuta Municipality. Among them, 9 schools (5 public and 4 private) were selected using a stratified random sampling technique. The public and private schools were considered as strata for selecting schools. The public and private schools consisted of 65% and 35% of the total students, respectively. Then, the required number of students from the public (n = 262) and private (n = 143) schools was calculated based on population proportionate sampling technique. A systematic random sampling technique was used to select participants. A first participant was randomly taken using the lottery method and every 4^th^ participant was taken in this study.

### Variables and operational definition

#### Adolescent girls

It is defined as the participants age between 10 and 19 years [27].

#### Types of anemia

It was classified into normal, mild, moderate and severe on the basis of hemoglobin level in the blood [21] (Table 1).

**Table 1:** Type of anemia based on hemoglobin level in the blood.

Type of anemia	10–11 years	12–19 years
	**g/dl**	**g/dl**
Normal	>10.4	>11.9
Mild	10.0–10.4	10.0–11.9
Moderate	7.0–9.9	7.0–9.9
Severe	<7.0	<7.0

Source: *The World Health Organization*

Table 1 describes the age-based classification of anemia based on the hemoglobin level in the blood.

Hemoglobin concentrations were adjusted for smokers (-0.3 g/dl) and those at elevations above 1000 meters (-0.2 g/dl) in this study [21].

#### Age

It was measured in completed years during the time of the study. Adolescents of age group “10–14” years as early adolescents and “15–19” years as late adolescents.

#### Ethnicity

The ‘advantaged’ group referred to Brahmin/Chhetri, while the ‘disadvantaged’ group referred to Janajati, Madhesi, Muslim, and Dalit.

**Religion** It was classified into Hindu, and non-Hindu (Kirat, Buddhist, Christian and Muslim).

#### Marital status

It was categorized into married and unmarried.

#### Education of mothers

It was grouped into illiterate (no education), primary level (below grade 8), secondary level (grade 8 to 12) and higher secondary level (higher than grade 12).

#### Normal menstrual cycle

It refers to the menstrual period of every 21–35 days [26].

#### Normal bleeding during menstruation

It was considered normal if it lasted for 2–5 days [26].

#### Pattern of skipping meal

If participants had skipped at least any of the meals (snacks/breakfast/lunch/dinner) for seven consecutive days [26].

#### Current smoker

If the participant had smoked within one month.

#### Current alcohol consumption

If the participant had taken alcohol within one month.

#### Barefoot

If participants did not wear shoes/slippers while working in the field.

#### Dietary diversity

Dietary diversity was assessed by the frequency (daily,7–10 times,3–6 times,1–2 times, not at all) of consuming eight food items i.e. fish, egg, milk, fruits, pulse, vegetable, cereals over the past seven days. Those answering at least once a week were coded as 1, and those giving the response of not taking were given the code 0, to each of the 8 items. The participant’s food score was computed as the sum of the responses to these questions, ranging from a lower of 0 to 8. A score above 5 was categorized as the presence of food diversity and a score between 0 to 4 was considered as the absence of food diversity [26,28].

#### Knowledge on “definition of anemia”

Participants were considered to have good knowledge if they correctly answered (Yes/No) “anemia is a decrease in the amount of Hb in the blood” [1].

#### Knowledge on signs/symptoms of anemia

Participants were considered to have good knowledge if they correctly answered (Yes/No) at least three relevant symptoms out of the mentioned symptoms.

#### Knowledge on preventive dietary foods of anemia

Participants were considered to have good knowledge if they correctly answered at least three iron-rich foods.

#### Body mass index (BMI)

It was categorized into underweight (<18.5 kg/m^2^), normal (18.5–24.9 kg/m^2^), and overweight (≥25 kg/m^2^) [26,29].

#### Stool RE (Routine Examination) /ME (Microscopic Examination)

Stool RE/ME evaluates stool for physical, chemical, and microscopic abnormalities, aiding in diagnosing infections or digestive disorders [30].

### Study tools

The study tool was adapted from the national survey on anemia among adolescent girls conducted in Nepal [20].

Participants’ height was measured with a stadiometer, and weight with a digital scale (SECA), recommended by UNICEF [31]. Blood and stool specimens were analyzed using a WHO recommended auto analyzer and microscope [9,30,32].

The pretesting of the tool was done among 10% of the similar population. Both Nepali and English translated language questionnaire was used to collect the information. A Cronbach alpha coefficient of 0.81 was obtained during the pretesting of study tool. Height and weight assessments were done two times per participants, and the average values were considered in this study [29].

### Data collection and techniques

A face-to-face interview using a semi-structured questionnaire was used to collect data from the participants. Each interview took around 30 minutes.

The principal investigator and research assistants were involved during the data collection. Research assistants were provided with orientation regarding the study project, data collection procedure, and handling of the instruments for data collection. Lab technicians and technologist were responsible for collecting and testing of blood and stool samples. Complete Blood Counts were assessed through a fully automated analyzer, and stool examinations were conducted using a microscope [9,30,32].

Height, weight, and blood samples of the participants were taken after completion of the interview. Stool samples were collected among the participants with hemoglobin levels <12 g/dl following the next day of data collection.

Anemic participants were provided with IFA supplementation as per the Government of Nepal (GoN) protocol. Anemia-focused health education programs were conducted in schools in collaboration with the school health teacher and the Health Section of Dhankuta Municipality.

### Data management and analysis

The collected data were entered, recorded, compiled, filtered, and cross-checked in Microsoft Excel and then exported into IBM Statistical Package for Social Sciences (SPSS) Version 25 for analysis. The descriptive results are presented in the form of frequency, percentage, mean, and standard deviation. The variables with p-value < 0.2 were included for the multivariable analysis to determine factors associated with anemia. All the tests were set at 95% Confidence Interval (CI) and p-value < 0.05 for statistically significant.

### Ethical approval

Ethical approval was obtained from the Institutional Review Committee (IRC) of B.P. Koirala Institute of Health Sciences (Reference number: 659/2023). A letter of support was obtained from Dhankuta municipality and the selected school authorities.

The purpose of the study was explained to participants and their parents. Written consent was taken from the parents/caregivers. Both verbal and written informed consent was obtained from the participants. Additionally, a written assent form was obtained from the participants who were below 18 years. Participant’s information was kept confidential, and anonymity was maintained. Laboratory test reports were made available to every participant.

## Results

### Social and demographical characteristics of participants

This study involved a total of 405 participants. The mean ± SD age was 14.2± 1.2 years. The majority of the participants were Janajati (63%) in ethnicity and Hindu (77.5%) in religion. Nearly two-thirds of the participants were from the government schools (64.7%). Around 60% of the participants belonged to nuclear families. Most of the participant’s mothers had completed secondary education (43.4%). Meanwhile, about 60% were involved in agriculture as their main occupation (Table 2).

**Table 2:** Social and demographical characteristics of participants.

Characteristics	Number (n)	Percentage (%)
**Age (Mean ± SD)**	**14.2 ± 1.2**	
10–14	220	54.3
15–19	185	45.7
**Ethnicity**		
Janajati	255	63
Brahmin/ Chhetri	119	29.3
Dalit	20	5.0
Madhesi	9	2.2
Muslim	2	0.5
**Religion**		
Hindu	314	77.5
Kirat	61	15.0
Buddhist	16	4.0
Christian	12	3.0
Muslim	2	0.5
**Marital status**		
Unmarried	404	99.8
Married	1	0.2
**Grade**		
8	100	24.7
9	156	38.5
10	149	36.8
**Type of school**		
Government school	262	64.7
Private school	143	35.3
**Type of family**		
Nuclear	242	59.8
Joint/extended	163	40.2
**Size of family**		
<5 member	202	49.9
≥5 member	203	50.1
**Education of mother (n = 401)**		
Illiterate	50	12.4
Secondary level	174	43.4
Primary level	121	30.2
Higher than secondary level	56	14.0
**Occupation of mother (n = 401)**		
Agriculture	244	60.8
Business	80	20.0
Government worker	46	11.5
Foreign employee	17	4.2
NGO/private	9	2.3
Daily labor	5	1.2
**Occupation of father (n = 395)**		
Business	108	27.3
Agriculture	96	24.3
Foreign employee	91	23.0
Government worker/officer	76	19.2
Daily labor	14	3.6
NGO/private	10	2.6

This table summarizes the demographic characteristics of the participants who participated in the study (Table 2).

### Status of anemia among participants

**Fig 1:**
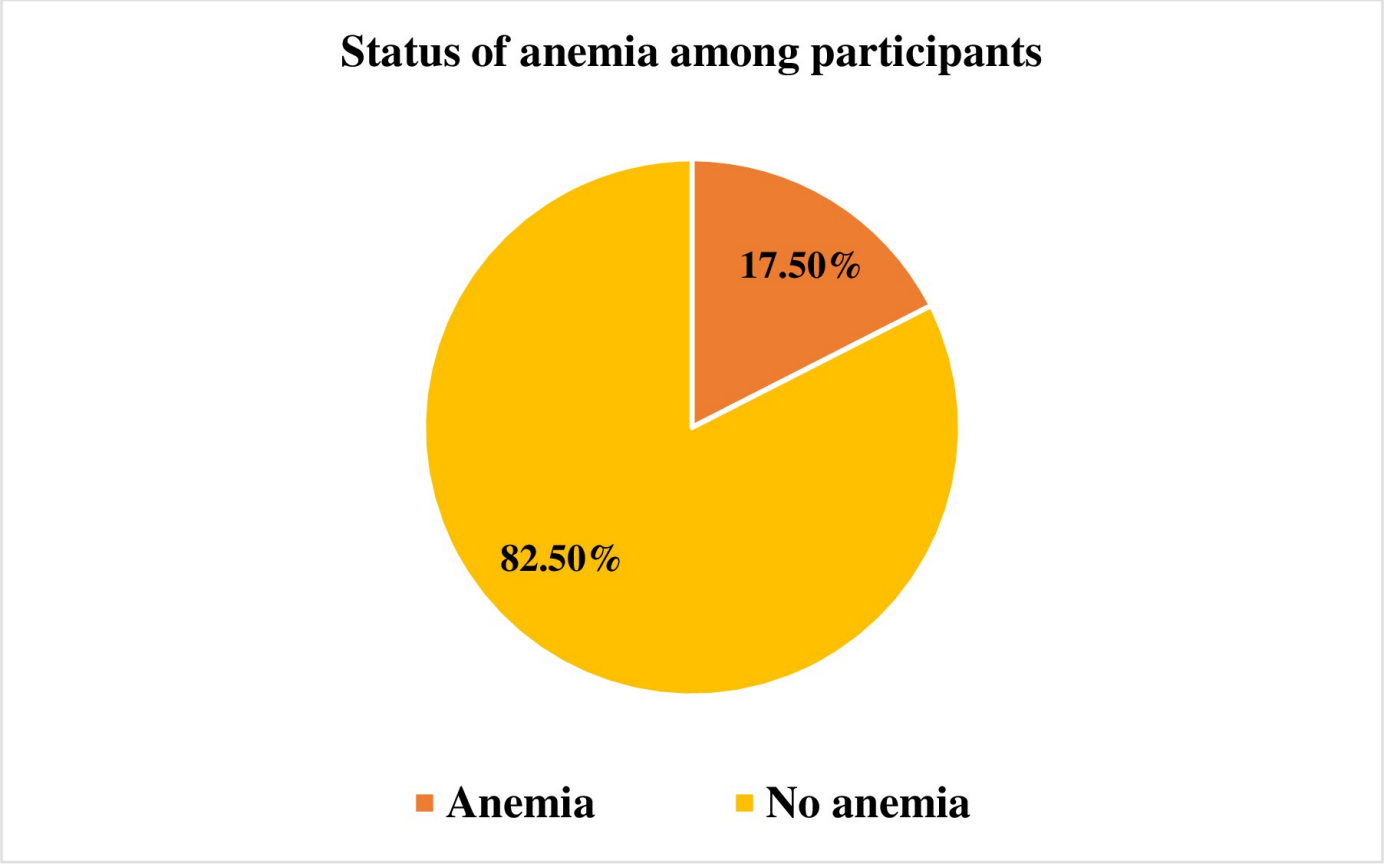
Prevalence of anemia among school going adolescents girls.

This study found that 17.5% (95% CI: 13.8, 21.2) of participants were anemic. Among them, 83.1% and 16.9% were mild and moderate anemia, respectively. The range of hemoglobin level was 8.1 g/dl to 15.1 g/dl (Fig 1).

### Reproductive health and behavioral-related characteristics

Most of the participants (95.3%) experienced menarche and 67.4% of participants had a normal menstrual cycle. Around one-third (67.1%) of the participants had normal bleeding during menstruation.

The majority (91.9%) of participants were non-vegetarians. Only 4.2% of participants had a history of chronic illness. Only 23.7% of participants reported a history of worm infestations within 2 years. While 41.2% of participants reported deworming within 6 months. Most of them, 85% scored the presence of food diversity. Half of the participants (55.6%) had taken iron and folic acid intake. The study showed that 13.1% 27.4%, and 59.5% had overweight, underweight, and normal BMI, respectively (Table 3).

**Table 3:** Reproductive health, behavioral, and BMI characteristics.

Characteristics	Number (n)		Percentage (%)
**Reproductive health**			
**Status of menarche**			
Yes	386		95.3
No	19		4.7
**Normal menstrual cycle (n = 386)**			
Yes	260		67.4
No	126		32.6
**Normal bleeding during menstruation (n = 386)**			
Yes	259		67.1
No	127		32.9
**Behavioral characteristics**			
**Drinking tea or coffee**			
Yes	181	44.7	
No	224	55.3	
**Pattern of skipping of meal**			
No	252	62.2	
Yes	153	37.8	
**Current smoker**			
No	401	99.0	
Yes	4	1.0	
**Current alcohol consumption**			
No	384	94.8	
Yes	21	5.2	
**Taking fresh lemon/pickles with food regularly**			
No	12	3.0	
Yes	393	97.0	
**Type of diet**			
Vegetarian	33	8.1	
Non-vegetarian	372	91.9	
**Times of junk food consumption (in a week)**			
Daily	177	43.4	
1–6 times	197	48.3	
7–10 times	34	8.3	
**History of chronic illness**			
Yes	17	4.2	
No	388	95.8	
**Main source of drinking water**			
Tap water	284	70.1	
Surface water	121	29.9	
**Purification of drinking water**			
Yes	352	86.9	
No	53	13.1	
**Frequency of washing hand (per day)**			
≥ 3 times	213	52.6	
< 3 times	192	47.4	
**Materials used for handwashing**			
Soap and water	400	98.8	
Only water	5	1.2	
**Using slippers/shoes while going outside**			
Yes	358	88.4	
No	47	11.6	
**History of worm infestation within 2 years**			
Yes	96	23.7	
No	309	76.3	
**Deworming within 6 months**			
No	238	58.8	
Yes	167	41.2	
**Food diversity (Mean± SD)**	6.1 ± 1.3		
Present	343	85.0	
Absent	62	15.0	
**Taking iron and folic acid**			
Yes	225	55.6	
No	180	44.4	
**Duration of iron and folic acid daily**			
≥ 26 weeks	175	77.8	
<26 week	50	22.2	
**BMI**			
Normal	241	59.5	
Underweight	111	27.4	
Overweight	53	13.1	
**Stool RE/ ME (n = 71)**			
Negative	65	91.5	
Presence of Entamoeba histolytica	6	8.5	

This table describes the reproductive health and behavioral-related characteristics of the participants in the study (Table 3).

### Dietary diversity pattern among participants

The majority of participants consumed meat, fruit, and cereals 1–2 times a day, 66.4% of participants consumed meat 1–2 times in the past week, while 69.5% did not consume fish (Table 4).

**Table 4:** Dietary diversity pattern in the last seven days.

Dietary Pattern	Meat (n = 372)	Fish (n = 371)	Egg (n = 389)	Milk (n = 405)	Fruit (n = 405)	Pulse (n = 405)	Vegetable(n = 405)	Cereals (n = 405)
	n (%)	n (%)	n (%)	n (%)	n (%)	n (%)	n (%)	n (%)
Daily	12 (3.2)	0 (0)	12 (3.1)	100 (24.7)	81 (20.0)	195 (48.1)	142 (35.1)	354 (87.4)
At least one time	302 (81.2)	113 (30.5)	284 (73)	227 (56)	294 (72.6)	173 (42.8)	247 (60.9)	48 (11.8)
Not at all	58 (15.6)	258 (69.5)	93 (23.9)	78 (19.3)	30 (7.4)	37 (9.1)	16 (4.0)	3 (0.7)

This table summarizes dietary diversity pattern among participants in the study. It includes consumption pattern of meat, fish, egg, milk, fruit, pulse, vegetable, and cereals within last seven days (Table 4).

### Knowledge of anemia among the participants

More than half i.e., 54% correctly identified the definition of anemia, 31% correctly reported signs/symptoms of anemia, and 24% were correctly defined the diets that prevents anemia.

### Factors association with anemia

Age, menarche status, menstrual cycle, type of diet, history of worm infestation, deworming and BMI were not associated with the prevalence of anemia in this study (Table 5).

**Table 5:** Factors associated with anemia among the participants.

Characteristics	Status of anemia		p-value	Odds Ratio (95% CI)
	Anemia n (%)	No anemia n (%)		
**Age**				
10–14	33 (15)	187 (85.0)		
15–19	38 (20.5)	147 (79.5)	0.144	1.4 (0.8,2.4)
**Status of menarche**				
No	1(5.3)	18 (94.7)		
Yes	70 (18.1)	316 (81.9)	0.219	3.9 (0.5,30.3)
**Normal menstrual cycle (n = 386)**				
Yes	47(18.1)	213 (81.9)		
No	23(18.3)	103 (81.7)	0.966	1.0 (0.5,1.7)
**Type of diet**				
Non vegetarian	62 (16.7)	310 (83.3)		
Vegetarian	9 (27.3)	24 (72.7)	0.125	1.8 (0.8,4.2)
**Taking fresh lemon/pickles regularly with food**				
Yes	68 (17.3)	325 (82.7)		
No	3 (25.0)	9 (75.0)	0.449	1.5 (0.4,6.0)
**Using slippers/shoes while going outside**				
Yes	64(17.9)	294(82.1)		
No	7(14.9)	40(85.1)	0.613	0.8 (0.3,1.8)
**History of worm infestation within 2 years**				
No	50 (16.2)	259 (83.8)	0.200	
Yes	21 (21.9)	75 (78.1)		1.4 (0.8,2.5)
**Deworming within 6 months**				
Yes	23 (13.8)	144 (86.2)		
No	48 (20.2)	190 (79.8)	0.960	1.5 (0.9,2.7)
**BMI**				
Normal	40 (16.6)	201 (83.4)		
Underweight	23 (20.7)	88 (79.3)	0.789	1.1 (0.4,2.5)
Overweight	8 (15.1)	45 (84.9)	0.349	0.7 (0.4,1.3)

This table describes the factors associated with the status of anemia among participants. It includes independent variables i.e. age, status of menarche, menstrual cycle, type of diet, history of worm infestation, intake of deworming, and BMI (Table 5).

### Multivariate logistic regression for factors associated with anemia among participants

The odds of having anemia was 3.3 times higher among the participants who did not correctly identify the definition of anemia (AOR = 3.3, CI: 1.0, 11.1). Similarly, participants who took IFA supplementation < 26 weeks had 26.9 times higher odds of having anemia (AOR = 26.9, CI: 8.3, 86.4). The participants not having food diversity had higher odds of having anemia (AOR = 2.5, CI: 1.1, 9.2) (Table 6).

**Table 6:** Multivariate logistic regression for factors associated with anemia.

Characteristics	Status of anemia		p-value	COR (95%CI)	p-value	AOR (95%CI)
	**Anemia** **n (%)**	**No anemia n (%)**				
**Pattern of skipping of meal**						
No	38 (15.1)	214 (84.9)		Ref		Ref
Yes	33 (21.6)	120 (78.8)	0.096	1.5 (0.9, 2.6)	0.282	1.7 (0.6, 5.0)
**History of chronic illness**						
No	65 (16.8)	323 (83.2)		Ref		Ref
Yes	6 (35.3)	11 (64.7)	0.049*	2.7 (0.9, 7.5)	0.736	0.6 (0.0, 6.7)
**Knowledge on definition of anemia**						
Correct	16 (7.3)	204 (92.7)		Ref		Ref
Incorrect	55 (29.7)	130 (70.3)	0.0001*	5.3 (2.9, 9.8)	0.046*	3.3 (1.0,11.1)
**Knowledge of symptoms of anemia**						
Correct	10 (7.9)	117 (92.1)		Ref		Ref
Incorrect	61 (21.9)	217 (78.1)	0.001*	3.2 (1.6, 6.6)	0.202	2.5 (0.6, 10.6)
**Knowledge of preventive food for anemia**						
Correct	9 (9.3)	88 (90.7)		Ref		Ref
Incorrect	62 (20.1)	246 (79.9)	0.014*	2.4 (1.1, 5.1)	0.290	0.4 (0.1, 1.9)
**Duration of taking iron and folic acid daily**						
≥ 26 weeks	11 (6.3)	164 (93.7)		Ref		Ref
< 26 weeks	26 (52.0)	24 (48)	0.0001*	16.2 (7.1, 36.8)	0.0001*	26.9 (8.3, 86.4)
**Junk food consumption (per week)**						
Not daily	17 (8.8)	177 (91.2)		Ref		Ref
Daily	54 (25.6)	157 (74.4)	0.0001*	3.5 (1.9, 6.4)	0.062	3.0 (0.9, 10.1)
**Dietary diversity**						
Present	42 (12.2)	301 (87.8)		Ref		Ref
Absent	29 (48.8)	33 (53.2)	0.0001*	6.3 (3.4, 11.4)	0.010*	2.5 (1.1, 9.2)

Ref.: Reference group, COR: Crude Odds Ratio, AOR: Adjusted Odds Ratio

*Statistically significant at the p-value <0.05.

A summary of factors associated with anemia among participants in the multivariate logistic regression analysis (Table 6).

## Discussion

Anemia among adolescents is a threat to low and middle-income countries like Nepal and becoming a significant public health concern, globally [3]. The objective of the study was to determine the prevalence of anemia and its associated factors among school-going adolescent girls.

This study found around 18% of the participants were anemic. Among them, 83.1% were mild and 16.9% were moderate. This finding is aligned with previous studies conducted among adolescent girls in Nepal i.e., 20% [9], India i.e., 20% [33]^,^ and Thailand i.e., 17% [3]. In contrast to this finding, a study conducted among developed countries such as Kuwait i.e., 10.9% [34], and Turkey i.e., 8.3% [35] reported a lower prevalence of anemia among adolescent girls. The prevalence in developed countries might be due to lower than in developing countries due to better nutrition, higher health literacy, improved healthy lifestyles, and socio-economic status [4]. Another reason is due to access to healthcare services, which results increase in detection and diagnosis of anemia [36]. This study showed a lower prevalence of anemia compared to previous studies, because government of Nepal have been conducting a biannual Iron Folic Acid supplementation (IFA) program routinely.

Additionally, iron-folic acid supplements and counseling on dietary diversity have been provided to adolescents through the school health program, aiming to prevent and control anemia.

The higher prevalence of anemia was reported among late adolescents compared to early adolescents. This finding is supported by the previous studies conducted in Nepal [26], India [37], and Kenya [38]. It is because of the onset of menstrual cycle among late adolescent girls which prompts physiological blood loss, inadequate dietary intake of iron to meet the increased nutritional demand [33]. Since the trend of early marriage and child bearing is higher among late adolescent in developing countries like Nepal [26]. This finding is supported by previous studies that reported higher prevalence of anemia among late adolescents [4,39].

This study found that the prevalence of anemia was higher among adolescent girls who had menarche and excessive blood loss during menstruation. Similar patterns were observed among study conducted in Nepal [20], India [37], Ethiopia [40] and Iran [41]. It is due to blood loss which is one of the major cause of anemia [42]. Adolescent girls who were vegetarian had 1.8 times higher odds to the risk of anemia than non-vegetarian adolescent girls. This findings is coherent with the similar studies conducted in Nepal [20], Kenya [43] and India [44]. It is because vegetarians and vegans are deficient in Vitamin B12 that results in low absorption of iron causing anemia [45].

Adolescent girls working without using slippers/shoes and having a history of hookworm infestation were at greater risk of anemia. This finding is comparable with the similar study conducted in South-Western Nepal [46]. It is due to hookworm infestation which can cause intestinal inflammation and damage, leading to poor absorption of iron, exacerbating the risk of anemia [26]. Similarly, it was reported that a lower prevalence of anemia among adolescent girls who were provided with deworming medication in the past 6 months. This finding is comparable with the similar studies conducted in the eastern part of Nepal, and Baglung district of Nepal [47,48]. Deworming reduces anemia by treating parasitic infections in gut and promote iron absorption [49].

The odds of having anemia was 1.08 times higher among adolescent girls who were underweight which is coherent with the other studies conducted in Nepal [47,48], India [50], and Lao PDR [51]. It may be due to factors such as poor nutrition intake and potentially compromised immune function, leading to increased vulnerability to infections [52].

This study showed that the prevalence of anemia was higher among adolescent girls who skipped their meals. Similar findings were reported from the study conducted in Ghana [53], Pakistan [54] and Indonesia [55]. It is due to low production of red blood cells resulted by low intake of nutrients such as iron among individuals who skip their meal [55].

Adolescent girls who had a history of chronic illness were at greater risk of anemia. This finding is consistent with a study conducted in the Mid-Western Terai Region of Nepal [20]. It may be due to factors such as underlying inflammation, impaired absorption of nutrients, medication side effects, and decreased production of red blood cells contribute to the risk of anemia [1].

A higher prevalence of anemia was found among adolescent girls with poor knowledge of anemia. It is because low health literacy leads to poor help-seeking behavior, delays in diagnosis of anemia, lack of awareness regarding preventive programs such as deworming and iron-folic acid supplementation, and poor preventive practices like habit of working with barefoot, and inadequate dietary habits, all these factors contribute to the risk of anemia [20,40,56].

Adolescent girls who took IFA supplements for at least 26 weeks had a lower prevalence of anemia compared to their counterparts. Similar findings were reported in the study conducted in Nepal [57], India [58], Kenya [43], and Vietnam [49]. This findings highlighted the effectiveness of IFA supplementation program to lower the risk of anemia among adolescents [9].

Consistent with other studies conducted in India [59], Indonesia [60], and Pakistan [61]. This study showed adolescent girls who consume junk food daily were at higher risk of anemia [59]. Junk foods are deficient in essential nutrients like vitamin C which results in low absorption of iron [61]. Additionally, high levels of processed sugar and unhealthy fats in junk food can interfere with nutrient absorption in the digestive tract, further reducing the body’s ability to absorb iron and other vital nutrients [61].

Dietary diversity plays a significant role in lowering the risk of anemia among adolescent girls. This study showed that lower prevalence of anemia among adolescent girls who had dietary diversity. These findings are aligned with a study conducted in Nepal [20], Ethiopia [52], Indonesia [55], and Nigeria [62]. Consuming a diverse range of foods increases the likelihood of obtaining an adequate intake of essential nutrients including iron, folate, vitamin B12, vitamin C, and other micronutrients crucial for red blood cell production and function [63]. A diverse diet helps ensure balanced nutrient intake that prevents the risk of anemia [64].

## Strengths and limitations

This study was conducted in rural areas including both public and private schools. The study investigated various characteristics such as socio-demographic, reproductive, dietary habits, behavioral, anthropometric measurements and biochemical tests that have a significant influence on anemia among adolescents.

There may be a chance of recall bias regarding dietary diversity, history of deworming, and junk food within 7 days. This study failed to consider a design effect while calculating sample size. Important factors like pregnancy, cultural and malarial infection were not taken in the study that could have a significant impact on the prevalence of anemia. Since the study is conducted in Dhankuta municipality i.e. hilly region, findings cannot be generalized to the adolescents residing in different geographical regions.

## Conclusion

This study revealed that 17.5% of adolescent girls were anemic. Higher prevalence of anemia were observed among older adolescent girls, those with limited knowledge about anemia, incomplete intake of IFA supplements, and lack of dietary diversity. These findings highlight the need for targeted interventions addressing adolescent girls’ nutritional knowledge, IFA supplementation, and dietary practices. Priorities should include school-based health programs, nutritional education, and promotion of dietary diversity for the prevention and control of anemia among adolescents.

## Supporting information

S1 File Participant information sheet. (DOCX)

S1 Questionnaire (DOCX)

S1 Data (XLSX)
