# Supplementary material for: Prevalence of anemia and its associated factors among school-going adolescent girls in schools of Dhankuta municipality, Nepal
Source: PLOS Glob Public Health. 2024 Sep 17;4(9):e0003684. doi: 10.1371/journal.pgph.0003684 (PMC11407645; doi:10.1371/journal.pgph.0003684)
Supplement: S1 File — (DOCX) [file pgph.0003684.s001.docx]

## Participant Informed Consent Form in English

**Protocol Number:** _______________

**Participant Identification number for the study:** ______________

**Title of the research:** “Prevalence of anaemia and its associated factors among adolescent girls studying in schools of Dhankuta municipality, Nepal”.

Name of the candidate: ________________, Age: ______years, Address ________________ Telephone: _____________ (mobile) _____________

(Residence/ parents) Email ________________

The content of the information sheet dated___________ that was provided, have been read carefully by me/explained in detail to me, in a language that I comprehend, and confirm that I have had the opportunity to ask questions. The nature and purpose of the study and its potential risks/ benefit and expected duration of the study, and other relevant details of the study have been explained to me in detail. I understand that my / my children’s participation is voluntary and that I am free to withdraw at any time, without giving any reason, without my medical care or legal right being affected.

I understand that the information collected about me/ my children from my participation in this research and sections of any of my medical notes may be looked at by responsible individuals from BPKIHS. I give permission for these individuals to have access to my record.

I hereby give consent to take part in the above study and allow to perform the procedure and any other medical service that may become necessary during the procedure.

I also consent for medical photographs/ video and I have been informed that these photographs/ video will be used without revealing the identity. I understand that these along with the information I provide may be used in my medical record, for purpose of publication in textbook or medical journal and dissertation purpose, or for medical education.

The consent form has been signed by me when I was not under the influence of any drugs.

Respondents/guardian signature ______________

Researcher’s signature ____________

Witness signature __________________

Date: ……………

**If the parents of an adolescent girls below 18 years are illiterate**

I have witnessed the accurate reading of the consent form to the potential participant and the individual has had the opportunity to ask questions and to understand the nature of study. I confirm that the individual has given consent freely. **Thumb print of participant**

**Right Left**

**Researcher’s signature ________________**

**Date:**

**Witness signature_________________**

## Participant Information Sheet in English

**Principal Investigator**: Milan Ghimire (Mobile Number 9852058844)

I, Milan Ghimire, an MPH student at the School of Public Health and Community Medicine at B.P. Koirala Institute of Health Sciences, Dharan. The research title was “Prevalence of Anaemia and Its Associated Factors among Adolescent Girls Studying in Schools in Dhankuta Municipality, Nepal.” I provided information and invited individuals to be a part of this research. Decision-making about participation was not required immediately; potential participants were encouraged to discuss the research with someone they trust before deciding.

I made sure to clarify any unfamiliar terms during the information sharing. If there were questions, I addressed them during or after the information session.

**Study Objectives:**

The study aimed to find out the prevalence of anaemia and its associated factors among school-going adolescent girls of Dhankuta Municipality.

**Study Duration:**

From June to October of 2023.

**Study Procedures and Protocol:**

The study involved teenagers aged 10-19 years. Data collection took place through direct interviews using a semi-structured questionnaire. Physical measurements of weight and height were taken, along with blood samples for hemoglobin measurement and stool samples to identify tapeworm presence. Interviews lasted approximately 20 to 25 minutes, while the collection of blood and stool samples required a maximum of one to two minutes.

**Role of Participants:**

Participants were asked about their social, demographic, reproductive health history, dietary patterns and behavior, and knowledge of anaemia. Additionally, a 2-milliliter blood sample was collected.

**Risks and harm:**

The study had minimal risks, with efforts made to minimize potential discomfort. Sensitive questions might have caused some participant’s distress. It was completely voluntary to skip any question or withdraw from the interview at any time.

**Benefits and Result Dissemination:**

Participants stood to benefit from this study. Apart from receiving education about anaemia prevention during adolescence, those with abnormal blood and stool test results were offered free treatment. Findings from this research would contribute to a thesis paper and could potentially be published in a scientific research journal.

**Privacy and Confidentiality:**

Confidentiality was a priority. Participants were assigned code numbers to maintain anonymity. Information collected was solely for research purposes, and names remained undisclosed.

**Voluntary Participation:**

Participation was entirely voluntary. Participants had the freedom to withdraw from the interview at any point without any issues. Choosing not to answer specific questions was also an option. Participation was based on personal preference.

**Contact Information:**

For queries, participants could reach out either at the time or even after the study had commenced:

Milan Ghimire

School of Public Health and Community Medicine

BPKIHS, Dharan, Nepal

Phone number: 9852058844

(Email ID: [ghmilan7@gmail.com](mailto:ghmilan7@gmail.com))

**Agreement to Participate:**

Do you agree to participate in this study?
