# Supplementary material for: Prevalence of anemia and its associated factors among school-going adolescent girls in schools of Dhankuta municipality, Nepal
Source: PLOS Glob Public Health. 2024 Sep 17;4(9):e0003684. doi: 10.1371/journal.pgph.0003684 (PMC11407645; doi:10.1371/journal.pgph.0003684)
Supplement: S1 Questionnaire — (DOCX) [file pgph.0003684.s002.docx]

## BP Koirala Institute of Health Sciences, Dharan, Nepal

School of Public Health and Community Medicine

Prevalence of Anaemia and Its Associated Factors among Adolescent Girls Studying in Schools of Dhankuta Municipality, Nepal

## Questionnaire for Data Collection in English

**Code no. Date:**

**School Name:**

|  | **Socio demographic Information:** | | | | | |
| --- | --- | --- | --- | --- | --- | --- |
| **SN** | **Questions** | | | **Response Code** | | |
| **Socio-Demographic Information** | | | | | | |
|  | Name of participant | | |  | | |
|  | Age of respondent (year completed) | | | ……Year | | |
|  | What is your caste/ethnicity? | | | 1. Dalit 2. Janajati 3. Madhesi 4. Mushlim 5. Bramhin/ Chhetri 6. Others (Specify) | | |
|  | What is your Religion? | | | 1. Hindu 2. Christian 3. Muslim 4. Buddhist 5. Others (Specify) | | |
|  | What is your marital status now? | | | 1. Married 2. Unmarried | | |
|  | Current Address | | | ……………………………… | | |
|  | Type of Family | | | 1. Nuclear 2. Joint/Extended   A7 | | |
|  | Family size in number | | | …………………… | | |
|  | Education of the respondent's mother | | | 1. No education 2. Primary level (up to 5 class) 3. Secondary level (5-12class) 4. Higher than Secondary level | | |
|  | Occupation of respondent’s mother | | | 1. Housewife 2. Agriculture 3. Government Worker /Officer 4. NGO/Private 5. Foreign Employee 6. Daily Labor 7. Business   Others (Specify) | | |
|  | Occupation of respondent’s father | | | 1. Unemployment 2. Agriculture 3. Government Worker /Officer 4. NGO/Private 5. Foreign employee 6. Daily Labor 7. Business 8. Others (Specify) | | |
|  | What is the main source of drinking water at your home? | | | 1. Tap water (public and private tap) 01 2. surface water (river, dams, ponds, streams, springs) | | |
| **History of reproductive health** | | | | | | |
|  | Are you menstruating/menarche? **If not, go to question number 18** | | | 1. Yes 2. No | | |
|  | If so, how old were you when you had your first menstrual period? | | | ………………………age | | |
|  | How is your menstrual cycle (21-35 days)? | | | 1. 21-35 days 2. Below 21 days 3. More than 35 days | | |
|  | How much number of days do you bleed? | | | 1. 2-5 days 2. More than 5 days | | |
| **Behavior and Food habit** | | | | | | |
|  | Do you treat your drinking water? **If no go to question number 20** | | | | 1. Yes 2. No | |
|  | What do you usually do to the water to make it safer to drink? **(Multiple response)** | | | | 1. Boiling 2. Water filter 3. Other (specify) | |
|  | When do you wash your hand? **(Multiple response)** | | | | 1. Before having meal 2. After having meal 3. After defecation 4. Others (specify) | |
|  | What do you used to wash your hand? | | | | 1. Water only 2. Soap and water both 3. Others (specify) | |
|  | Do you wear shoes while going outside home? | | | | 1. Yes 2. No | |
|  | Do you drink tea or coffee every day? **If no go to question no. 25.** | | | | 1. Yes 2. No | |
|  | Do you especially drink tea or coffee within 2 hours of meal? | | | | 1. Yes 2. No | |
|  | In a typical week, are you skipping any meal? **If No, go for the question no.28** | | | | 1. Yes 2. No | |
|  | If you have stopped eating, what foods have you stopped eating regularly? **(Multiple Response)** | | | | 1. Breakfast 2. Lunch 3. Day snacks 4. Dinner 5. Other (Specify) | |
|  | What is the main reason for skipping meal? **(Multiple Response)** | | | | 1. Due to food deficient 2. For weight loss 3. For attractive figure 4. Health conscious 5. No time to have meal 6. By not liking food 7. Other (Specify) | |
|  | Are you currently smoking? | | | | 1. Yes 2. No | |
|  | Are you currently drinking alcohol? | | | | 1. Yes 2. No | |
|  | Does anyone in your family currently drink alcohol/drugs? | | | | 1. Yes 2. No | |
|  | Do you consume lemon or fresh pickle along with your food? | | | | 1. Always do 2. I do sometimes 3. I don't | |
|  | Do you currently have any chronic illnesses? **If No, go to the question no.34** | | | | 1. Yes 2. No | |
|  | If yes, which chronic disease? | | | | .................................................. | |
| **General knowledge about anaemia** | | | | | | |
|  | What is anaemia? | | 1. The level of hemoglobin/iron is lower than normal 2. The amount of white blood cells is less than normal in blood 3. Don’t know | | | |
|  | What are the signs and symptoms of anaemia?  **(Multiple response)** | | 1. Easy fatigue and loss of energy 2. Unusually rapid heartbeat 3. Shortness of breath 4. Headache 5. Difficulty concentrating 6. Dizziness 7. Pale skin 8. Leg cramps 9. Insomnia 10. Don’t know | | | |
|  | What are the names of the foods that should be eaten to avoid anaemia? **(Multiple response)** | | 1. By eating green leafy vegetables 2. By eating legumes 3. By eating barley 4. By eating ground food 5. By eating organ meat, blood, liver 6. By eating food cooked iron vessels 7. Don’t know | | | |
|  | Are you taking iron/folic acid? **If no go to Question No. 40** | | 1. Yes 2. No | | | |
|  | If yes, when and how you started? | | …………………………………………………………………………… | | | |
| **Dietary Diversity**  Now I would like to ask you about frequency of liquids or foods that you have eat or drink in the last 7 days. | | | | | | |
|  | How is your eating habit?  **If Eggitarian go to Question No. 43 and if Vegetarian go to Question No. 44.** | | | | | 1. Vegetarian 2. Eggitarian 3. Non vegetarian |
|  | How many times a week did you eat meat (liver, blood, red meat, sausage)? | | | | | 1. Daily 2. 7-10 times 3. 3-6 times 4. 1-2 times 5. Was not eat |
|  | How many times a week did you eat fish (shellfish, prawns, shidra and other fish)? | | | | | 1. Daily 2. 7-10 times 3. 3-6 times 4. 1-2 times 5. Was not eat |
|  | How many times did you eat eggs in a week? | | | | | 1. Daily 2. 7-10 times 3. 3-6 times 4. 1-2 times 5. Was not eat |
|  | How many times have you eaten dairy products (yogurt, honey, milk, paneer, curd, etc.) in a week? | | | | | 1. Daily 2. 7-10 times 3. 3-6 times 4. 1-2 times 5. Was not eat |
|  | How many times a week did you consume any fruit and juice (pomegranate, beetroot, sarifa mango, peach, lychee, apple etc.)? | | | | | 1. Daily 2. 7-10 times 3. 3-6 times 4. 1-2 times 5. Was not eat |
|  | How many times a week did you consume any pulses (red lentils, black lentils etc.), soybeans, Bengal gram? | | | | | 1. Daily 2. 7-10 times 3. 3-6 times 4. 1-2 times 5. Was not eat |
|  | How often did you consume any dark green leafy vegetables such as spinach, pumpkin leaves, lettuce leaves, banda govi, gundruk, or leaves, beans, green beans, beet greens, garlic, mustard greens, spinach, turnip leaves, peach leaves, leaves of skush in a week? | | | | | 1. Daily 2. 7-10 times 3. 3-6 times 4. 1-2 times 5. Was not eat |
|  | How many times in a week did you eat the following food items (rice, maize, millet, wheat, yam, chapati, roti, thukpa, dalia, etc.)? | | | | | 1. Daily 2. 7-10 times 3. 3-6 times 4. 1-2 times 5. Was not eat |
|  | Do you eat junk food or processed foods? If yes, how many times in a week did you eat the following items such as chips, Kurkure, Lage, noodles (chauchau), carbonated drinks coke, Fanta, cheeseballs, salty or cookies biscuits etc.? | | | | | 1. Daily 2. 7-10 times 3. 3-6 times 4. 1-2 times 5. Was not eat |
| **Others** | | | | | | |
|  | Have you ever been suffered from worm infestation? (**If no go to question no.51)** | | | | | 1. Yes 2. No |
|  | If you have had a worm infestation, when did it happen? | | | | | 1. Within this 6 month 2. Within 6-12 month 3. More than 1 year |
|  | Did you take drugs for intestinal worms within last 6 months? | | | | | 1. Yes 2. No |
| **Anthropometric measurement** | | | | | | |
|  | | Weight 1 | | | | ……….kg |
|  | | Weight 2 | | | | ……….kg |
|  | | Average Weight | | | | ……….kg |
|  | | Height 1 | | | | …………m |
|  | | Height 2 | | | | …………m |
|  | | Average Height | | | | …………m |
|  | | BMI | | | | ………. Kg/m2 |
| **Biochemical test** | | | | | | |
|  | | Hemoglobin level (gm/dl) | | | | …………gm/d |
|  | | Stool RE/ME | | | | ……………….. |
| Interviewer’s signature | | | | | | ……………………… |
| Respondent’s signature | | | | | | …..…………………… |

**“Thank you for your valuable time and participation”**
